# A Mendelian randomization study on causal effects of inflammatory bowel disease on the risk of erectile dysfunction

**DOI:** 10.1038/s41598-024-52712-1

**Published:** 2024-01-25

**Authors:** Di Chen, Chao Zhou, Quanhai Luo, Changsheng Chen, Gang Liu

**Affiliations:** 1 Department of Urology, Reproductive Hospital of Guangxi Zhuang Autonomous Region, Nanning, China; 2 Department of Urology, The Frist Affiliated Hospital of Guangxi Medical University, Nanning, China; 3 Department of Assisted Reproduction, Nanxishan Hospital of Guangxi Zhuang Autonomous Region, Guilin, China; 4 Department of Urology, People’s Hospital of Guangxi Zhuang Autonomous Region, Nanning, China

**Keywords:** Gastroenterology, Urology

## Abstract

This study aimed to evaluate the causal effects of inflammatory bowel disease (IBD) and erectile dysfunction (ED) using Mendelian randomization (MR). All datasets were obtained from the public genome-wide association study database. In the exposure group, 12,882 IBD patients and 21,770 controls were included. A total of 1154 ED patients and 94,024 controls were included in the outcome group. Two-sample MR was conducted to estimate the causal effect of IBD on ED. Furthermore, Crohn's disease (CD) and ulcerative colitis (UC) were exposure factors in subgroup analyses. Weighted median, MR-egger, Inverse-variant weighted (IVW), weighted mode, and simple mode methods were used in MR analysis. Horizontal pleiotropy test, heterogeneity test, and leave-one-out method were utilized to evaluate the sensitivity and stability of results. After analysis, 62, 52, and 36 single nucleotide polymorphisms (SNPs) that IBD-ED, CD-ED, and UC-ED were included, respectively. The incidence of ED was increased by IBD (IVW: OR = 1.110, 95% CI = 1.017–1.211, *P* = 0.019; P-heterogeneity > 0.05) and, in addition, ED was affected by CD (IVW: OR = 1.085, 95% CI = 1.015–1.160, *P* = 0.016; P-heterogeneity > 0.05). However, there was no causal effect of UC on ED (IVW: OR = 1.018, 95% CI = 0.917–1.129, *P* = 0.743; P-heterogeneity < 0.05). All SNPs showed no significant horizontal pleiotropy (*P* > 0.05). These results indicate that IBD and CD can cause ED; However, UC did not cause ED. Additional research was required to determine causality and potential mechanisms further.

## Introduction

Inflammatory bowel disease (IBD) is a chronic and non-specific gastrointestinal tract inflammatory disease including two major clinical subtypes, Crohn's disease (CD) and ulcerative colitis (UC), usually diagnosed in young individuals between 15 and 40 years old^1,2^. In Western Europe, the reported incidence of CD ranges from 1.85 to 10.6 per 100,000 person-years; for UC, the numbers are 1.9 to 17.2 per 100,000 person-years^3^. CD causes chronic inflammation in all parts of the intestinal tissue along the entire gastrointestinal tract, while UC is limited to the mucosa of the colon and rectum. Patients with IBD suffer from diarrhea, abdominal pain, weight loss, and various extraintestinal manifestations^4^. Due to persistent symptoms or acute exacerbations, IBD significantly impacts health-related quality of life. Several studies have found that IBD also affects the sexual function of patients. Timmer et al. performed a case–control study and presented that 44% of male patients with IBD reported a severely compromised sexuality^5^. Similarly, a retrospective study by Kao et al. found that the risk of erectile dysfunction (ED) in patients with IBD was 1.64 times higher than in normal men after matching age, comorbidities, and medication factors^6^. However, the study of Valer et al. found no significant relationship between sexual dysfunction and IBD, but 92% of included IBD patients were in clinical remission^7^. Whether the association between IBD and ED reflects a causal relationship remains unclear.

ED is the persistent inability to obtain or maintain a sufficient penile erection for satisfactory sexual intercourse. It is a common male sexual dysfunction caused by multiple factors^8^. Traditionally, owing to microvascular and testosterone level changes in older men, ED has been considered an aging male disease^9^. Recently, a large multinational study reported that the prevalence of ED in young men is as high as 30%^10^. Depression, anxiety, and organic lesions are leading causes of ED. A relationship also exists between chronic disease and ED. Hypertension and diabetes are the most common chronic diseases that cause ED. IBD occurs in young people at the peak of sexual activity, and the association with ED has attracted much attention. O’Toole et al. found that male sexual dysfunction in IBD patients was positively linked to depression, an ileoanal pouch anastomosis, and active disease^11^. Park et al. performed a review and suggested that surgery and medical therapy may contribute to sexual difficulties in IBD patients^1^. According to the European Society for Parenteral and enteral nutrition guidelines, poor nutritional status is prevalent in IBD patients and might cause sexual difficulties^12^. Although multiple factors have proposed for the development of ED in IBD patients, conclusions based on observational studies are may be influence by confounding factors and do not elucidate the causal relationship between diseases. A large-sample randomized controlled study is needed to explore the causal relationship between IBD and ED.

Mendelian randomization (MR) is a widely used epidemiological method for making causal inferences of clinical exposures on explicit outcomes based on the instrumental variable^13^. Unlike randomized controlled trials, MR can eliminate the effects of confounding factors and reverse causality while avoiding a huge amount of time and economic consumption. Single nucleotide polymorphisms (SNPs) are often used as instrumental variables in MR^14^. SNPs are single nucleotide site variants in the genome and are randomly assigned among each individual^15^. With the advantages of genetic stability, SNPs are free from the environment, time, and lifestyle. Genome-wide association studies (GWAS) provide information on multiple disease-associated SNPs based on large patient populations. Our study proposed IBD was used as an exposure factor and ED as an outcome factor. Based on GWAS data, MR analyses are performed to investigate whether IBD can cause the development of ED.

## Materials and methods

### Study design

**Figure 1:**
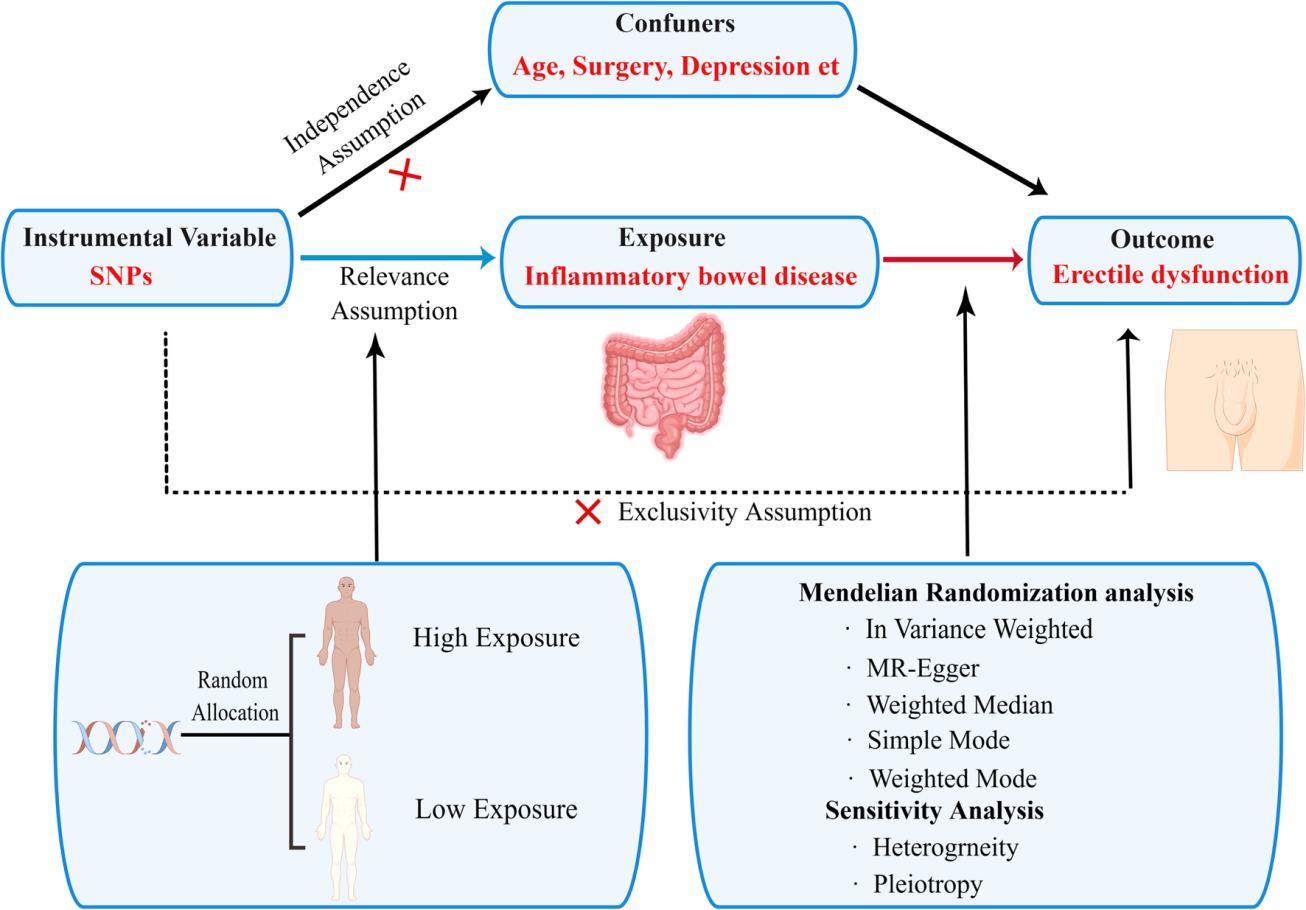
A flow chart illustrating the causality of the relationship between inflammatory bowel disease and erectile dysfunction.

The present study used IBD (including CD and UC) as exposure factors, while ED was the outcome factor for MR analysis. Based on mutual SNPs, the effect of IBD on ED risk was calculated. The analysis was based on the following assumptions: (1) the genetic instrumental variable was associated with the exposure; (2) the genetic instrumental variable was not associated with confounders; (3) the genetic instrumental variable influences the outcome factor only by the exposure factor. Figure 1 demonstrates the study design.

### Data sets

IBD and ED datasets obtained from GWAS, including openGWAS, IEU, and GWAS Catalog, were searched and downloaded as eligible datasets. The IBD dataset contained 12,882 cases and 21,770 controls for the European population (SNP = 12,716,084, ID: ieu-a-31). The CD dataset contained 5956 cases and 14,927 controls for the European population (SNP = 12,276,506, ID: ieu-a-30). The UC dataset contained 6,968 cases and 20,464 controls for the European population (SNP = 12,255,197, ID: ieu-a-32). The ED dataset contained 1154 cases and 94,024 controls for the European population (SNP = 16,378,833, ID: finn-b-ERECTILE_DYSFUNCTION).

The SNPs were selected based on the following criteria: (1) the SNP with significant association with IBD or its subtype (*P* < 5 × 10^−8^); (2) the SNP with significant association with ED (*P* < 5 × 10^−8^); (3) SNPs without a linkage disequilibrium (R^2^ > 0.001 within a 10,000 kb window). Two-sample MR in the R package was utilized for data selection and Mendelian Randomization analyses.

### Mendelian randomization

After harmonization, selected SNPs were instrumental variables. The causal effect of IBD and substyle on ED was mainly evaluated by the standard inverse variance weighted method; the weighted median, MR-Egger, weight mode methods, and simple mode as supplementary analysis were also performed. The slope of the MR-Egger regression provided the estimate of the causal association between the exposure factor and outcome factor. If the intercept in MR-Egger regression were truly zero (or were constrained to be zero), the SNP outcome effects were mediated solely through the exposure factors.

Sensitivity analyses were performed to test for heterogeneity and horizontal pleiotropy. Cochrane’s Q statistics evaluated the heterogeneity of the inverse variance weighted and MR-Egger methods. If there was significant heterogeneity (*P* < 0.05), the results were summarized using a random-effects inverse variance weighted method. Otherwise, a fixed-effect inverse variance weighted method was applied. Horizontal pleiotropy was evaluated by MR-Egger regression using mr_pleiotropy_test with a p-value threshold of 0.05. In addition, the leave-one-SNP- out sensitivity method was adopted to identify whether the causality effect was affected by a single SNP under the inverse variance weighted method.

## Results

### ***SNPs ***selection

After analysis, 62 SNPs related to IBD and ED were included. Then, 52 selected SNPs were related to CD and ED. Furthermore, 36 selected SNPs were related to UC and ED.

All selected SNPs were treated as instrumental variables and met the exposure genome-wide significance criteria (*P*<5×10^−8^, r^2^<0.001, kb=10,000).

### Mendelian randomization estimation between IBD and ED

**Figure 2:**
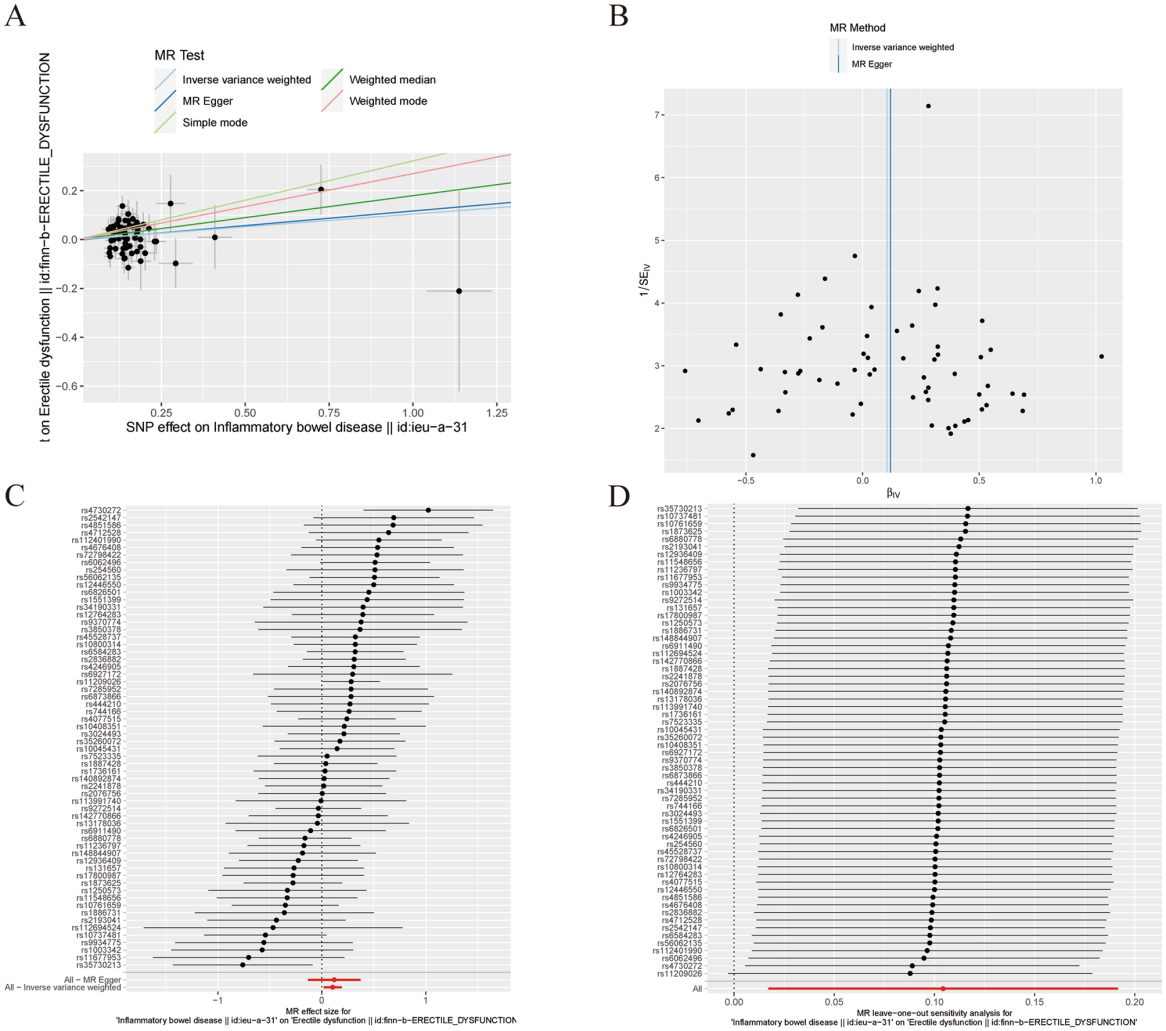
Association between Inflammatory bowel disease and risk of erectile dysfunction. (**A**) multiple MR tests showed the SNP effects; (**B**) funnel plot for Inflammatory bowel disease risk of ED; (**C**) effect size of each SNP funnel plot for Inflammatory bowel disease risk of ED; (**D**) leave-one-out sensitivity analysis.MR Mendelian Randomization, SNP single nucleotide polymorphism, ED erectile dysfunction.

MR analysis demonstrated that IBD may contribute to ED. The results were consistent with weighted median (OR=1.197, 95% CI=1.059–1.353, *P*=0.004), inverse variance weighted (OR=1.110, 95% CI=1.017–1.211, *P*=0.019), weighted mode (OR=1.310, 95% CI=1.037–1.654, *P*=0.027), simple mode (OR=1.380, 95% CI=1.038–1.654, *P*=0.027) (Table 1, Figure 2A).

**Table 1:** The association of inflammatory bowel disease with Erectile dysfunction outcomes by Mendelian randomization analysis.

MR method	Number of SNPs	Beta	SE	OR	95% confidence interval	Association *P*-value	Cochran Q statistic	Heterogeneity *P*-value
MR Egger	62	0.120	0.129	1.127	0.875–1.452	0.359	72.871	0.123
Weighted median	62	0.180	0.062	1.197	1.059–1.353	0.004	–	–
Inverse variance weighted	62	0.104	0.045	1.110	1.017–1.211	0.019	72.890	0.141
Simple mode	62	0.322	0.145	1.380	1.038–1.835	0.030	–	–
Weighted mode	62	0.270	0.119	1.310	1.037–1.654	0.027	–	–

The heterogeneity analysis highlighted that no significant heterogeneity was detected in the inverse variance weighted and MR-Egger methods (*P* > 0.05). The horizontal pleiotropy test suggested no significant horizontal pleiotropy was detected in the MR-Egger method (*P* = 0.901). The funnel plot was symmetric, so no horizontal pleiotropy was observed (Fig. 2B). Figure 2C plot shows the effect size of each SNP. In addition, the leave-one-SNP-out analysis found that the estimated association was not disproportionately influenced by a single SNP (Fig. 2D).

### Mendelian randomization estimation between CD and ED

**Figure 3:**
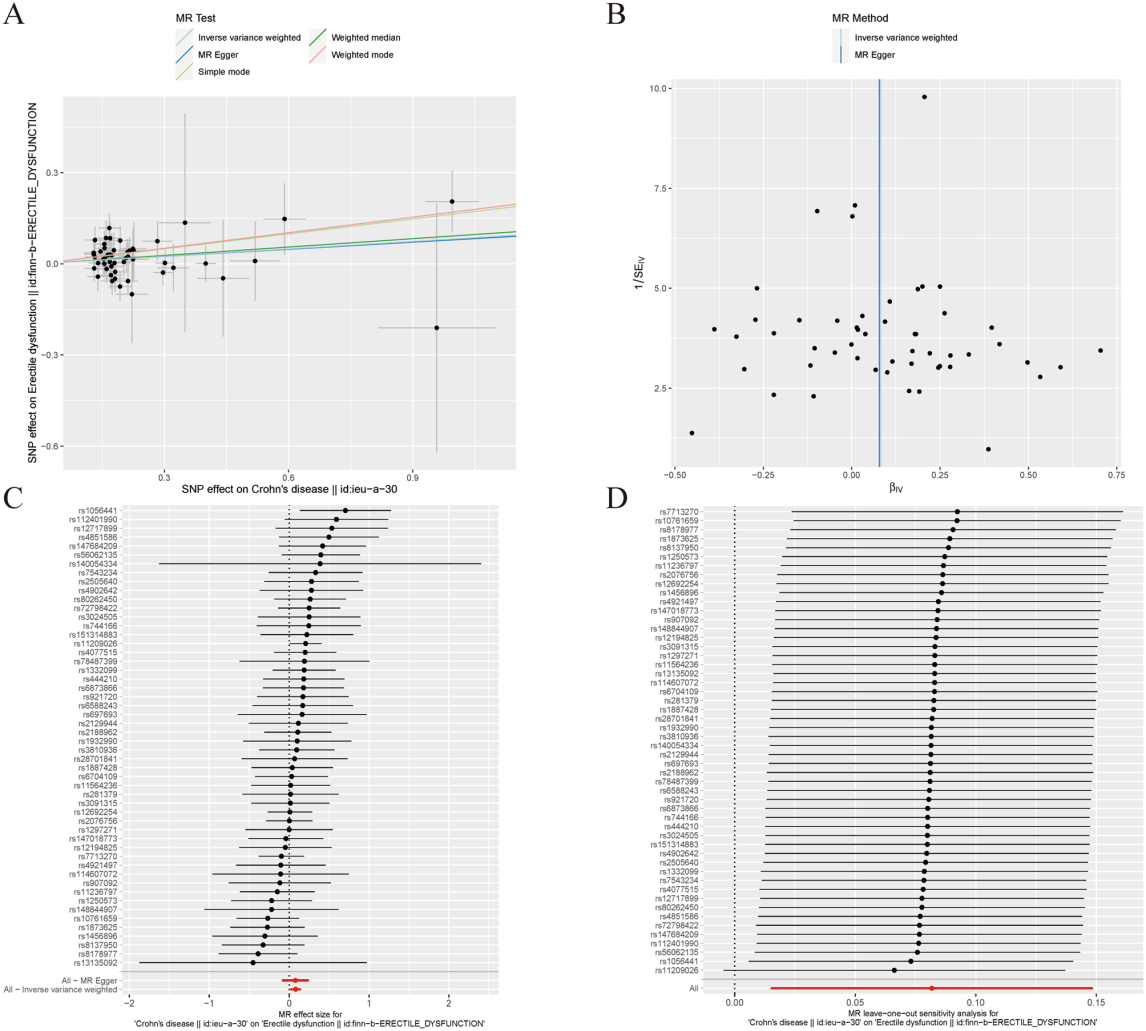
Association between Crohn's disease and risk of erectile dysfunction. (**A**) multiple MR tests showed the SNP effects; (**B**) funnel plot for Crohn’s disease risk of ED; (**C**) effect size of each SNP funnel plot for Crohn's disease risk of ED; (**D**) leave- one-out sensitivity analysis.MR Mendelian Randomization, SNP single nucleotide polymorphism, ED erectile dysfunction.

Our MR analysis demonstrated that CD may contribute to ED. The results were consistent with inverse variance weighted (OR=1.085, 95% CI=1.015–1.160, *P*=0.016), weighted mode (OR=1.186, 95% CI=1.007-1.397, *P*=0.046) (Table 2, Figure 3A).

**Table 2:** The association of Crohn's disease with Erectile dysfunction outcomes by Mendelian randomization analysis.

MR method	Number of SNPs	Beta	SE	OR	95% confidence interval	Association *P*-value	Cochran Q statistic	Heterogeneity *P*-value
MR Egger	52	0.078	0.085	1.081	0.915–1.277	0.364	39.371	0.860
Weighted median	52	0.092	0.051	1.096	0.992–1.212	0.072	–	–
Inverse variance weighted	52	0.082	0.034	1.085	1.015–1.160	0.016	39.373	0.882
Simple mode	52	0.164	0.103	1.178	0.963–1.442	0.117	–	–
Weighted mode	52	0.171	0.084	1.186	1.007–1.397	0.046	–	–

The heterogeneity test highlighted that no significant heterogeneity was detected in the inverse variance weighted and MR-Egger methods (*P* > 0.05). The horizontal pleiotropy test suggested no significant horizontal pleiotropy was detected in the MR-Egger method (*P* = 0.962). The funnel plot was symmetric, so no horizontal pleiotropy was observed (Fig. 3B). Figure 3C plot shows the effect size of each SNP. In addition, the leave-one-SNP-out analysis found that the estimated association was not disproportionately influenced by a single SNP (Fig. 3D).

### Mendelian randomization estimation between UC and ED

**Figure 4:**
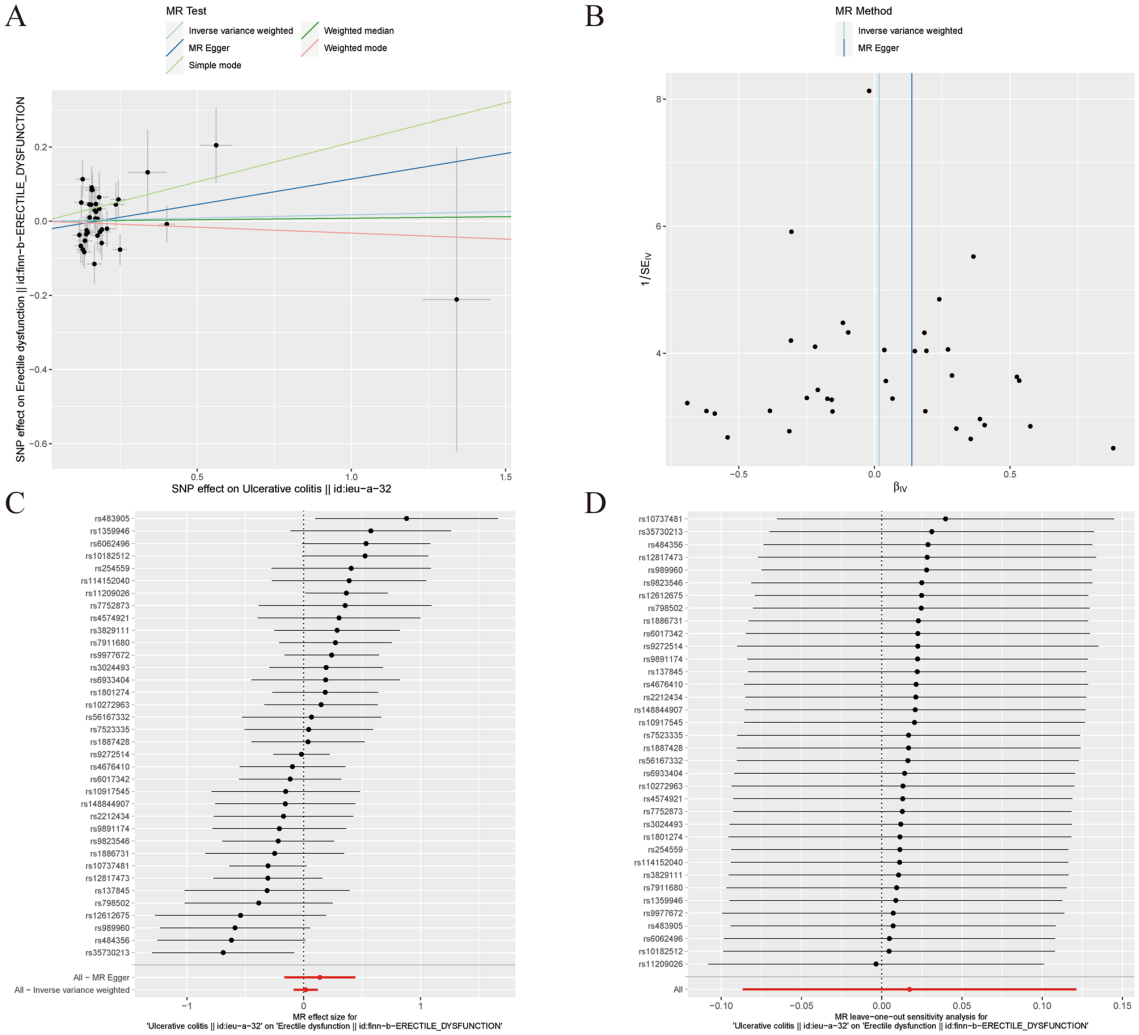
Association between ulcerative colitis and risk of erectile dysfunction. (**A**) multiple MR tests showed the SNP effects; (**B**) funnel plot for ulcerative colitis risk of ED; (**C**) effect size of each SNP funnel plot for ulcerative colitis risk of ED; (**D**) leave- one-out sensitivity analysis. MR Mendelian Randomization, SNP single nucleotide polymorphism, ED erectile dysfunction.

Our MR analysis demonstrated that UC might not contribute to ED. The results were consistent with weighted median (OR=1.008, 95% CI=0.878–1.158, *P*=0.907), inverse variance weighted (random effects) (OR=1.018, 95% CI=0.917–1.129, *P*=0.743), weighted mode (OR=0.968, 95% CI=0.772–1.214, *P*=0.783), simple mode (OR 1.237, 95% CI 0.907–1.688, *P*=0.188) (Table 3, Figure 4A)

**Table 3:** The association of Ulcerative colitis with Erectile dysfunction outcomes by Mendelian randomization analysis.

MR method	Number of SNPs	Beta	SE	OR	95% confidence interval	Association *P*-value	Cochran Q statistic	Heterogeneity *P*-value
MR Egger	36	0.138	0.155	1.148	0.847–1.556	0.381	51.989	0.025
Weighted median	36	0.008	0.071	1.008	0.878–1.158	0.907	–	–
Inverse variance weighted (fixed effects)	36	0.017	0.043	1.018	0.935–1.107	0.687	53.033	0.026
Inverse variance weighted (random effects)	36	0.017	0.053	1.018	0.917–1.129	0.743	53.033	0.026
Simple mode	36	0.212	0.159	1.237	0.907–1.688	0.188	–	–
Weighted mode	36	−0.032	0.115	0.968	0.772–1.214	0.783	–	–

The heterogeneity test highlighted that significant heterogeneity was detected in the inverse variance weighted and MR-Egger methods, respectively (*p* = 0.026, *P* = 0.025). The horizontal pleiotropy test suggested no significant horizontal pleiotropy was detected in the MR-Egger method (P = 0.414). The funnel plot was symmetric, so no horizontal pleiotropy was observed (Fig. 4B). Figure 4C plot shows the effect size of each SNP. In addition, the leave-one-SNP-out analysis found that the estimated association was not disproportionately influenced by a single SNP (Fig. 4D).

## Discussion

Our two-sample MR analyses suggested that IBD causes an increased risk of ED. Further, IBD subtype analysis found that CD may cause an increased risk of ED, whereas UC may not lead to an increased risk of ED. Sensitive analysis revealed that our results were stable. Heterogeneity analysis suggested no significant heterogeneity in the IBD and CD. In the UC subgroup analysis, we observed that significant heterogeneity existed. An inverse variance weighted (random-effect model) was performed and showed UC might not cause an increased risk of ED. Also, SNP pleiotropy tests found that no significant horizontal pleiotropy was present.

Limited research focuses on the correlation between IBD and ED. Several studies suggested that IBD patients with a higher prevalence of ED. Zhang et al. performed a cross-sectional study that included 208 IBD patients and 190 normal individuals assessed ED by the international index of erectile function^16^. The results found that IBD patients had a threefold higher prevalence of ED than normal individuals, and depression was an independent risk factor for ED. A similar study by Domislovic et al. also found that the incidence of ED was as high as 30.3% in IBD patients, and depression was a significant predictor of ED in IBD patients^17^. Instead, Bel et al. performed a cross-sectional study that included 119 IBD male patients and found no significant ED risk difference between the IBD patient group and the healthy men control group^18^. These cross-sectional studies reported a higher incidence of ED among IBD patients, suggesting some correlation between IBD and ED. However, the direction of the association cannot be established in cross-sectional studies (causes and effects). Shmidt et al. performed a prospective single-arm cohort study that included 69 IBD patients with a minimum two-year follow-up^19^. They found that 94% of IBD patients were diagnosed with ED, and poor physical and mental well-being were vital risk factors for ED. The prospective randomized controlled study is commonly used to explore the causal direction of the association, while long-term and strict follow-up is needed. Kao et al. performed a large sample size cohort study that included 1845 IBD patients and an age-matched control group^6^. After follow-up, they found a 1.6-fold ED risk increase in the IBD patients group compared to the control; the abnormal psychological factor is the leading cause of ED. Confounding factors confounded the results of this retrospective cohort study that still did not reveal a causal relationship between IBD and ED. However, no prospective randomized control trials have been conducted to address the relationship between IBD and ED. Our study is the first MR study exploring the causal relationship between IBD and ED, which may aid in further understanding the sexual activity in IBD patients.

ED development and progression is a complicated process affected by various etiological factors. Psychological factors, such as depression and anxiety, may cause ED by negative interaction and recognition^20^. Ma performed an MR study and found that depression increases ED risk^21^. Similarly, an MR study by Chen et al. found that patients with major depression may develop ED, but bipolar disorder did not cause ED^22^. These studies were unidirectional MR analyses, and ED may also cause depression. A case–control study by Manalo et al. found that young ED patients had a high depression risk before and after ED diagnosis^23^. Like other chronic illnesses, IBD also has an impact on psychological health. Depression is prevalent in IBD patients, of which 14.9% reported having major depression^24^. We hypothesized that depression may be a mediator for ED during IBD. In addition, IBD medications may cause ED. Several cases of sulfasalazine-induced ED have been reported^25^. Within a 10-year observation period, the cumulative surgery rate was 20–50% in patients diagnosed with CD and 10% in those diagnosed with UC^26^. Wu et al. performed a meta-analysis and found that surgery increased the risk of ED in IBD patients^20^. In the study of wang et al., the authors believed that surgery was beneficial to health improvement in IBD patients^27^. Rectal surgery and stoma are standard surgical procedures for IBD. However, rectal surgery may damage pelvic nerves and alter anatomy, causing ED. Stoma patients obtained lower sexual satisfaction due to body image^28^. A rat model study by Villegas et al. found that nerve-sparing pelvic surgery also caused a high risk of ED because of the systemic inflammatory response^29^. Based on the advantages of MR, our study avoided the effects of confounding factors such as age, treatment, and mental state.

IBD may cause penile organic lesions leading to ED. A study by Brescia et al. found that IBD can cause intestinal vascular barrier dysfunction that permits entry of microorganism products and pro-inflammatory factors, which lead to low-grade systemic vascular inflammation and impairment of systemic vasculature barriers^30^. Many studies found a relationship between IBD and cardiovascular events^31–35^. Increasing circulating inflammatory cytokines IL-6 and TNF-α were found in IBD patients and animal models^36^. IL-6 directly affected the activity and expression of nitric oxide synthase in vascular endothelial cells^37^. In addition, IBD patients were found at a high risk of thrombotic events^38,39^. Compared to other organs, the cavernous arteries of the penis are smaller in diameter and have a high endothelial cell content per gram of tissue. Therefore, ED may be an early clinical manifestation of systemic vascular disease^40^. IBD causes microvascular endothelial dysfunction with nitric oxide-dependent loss of dilation, poor wound healing, persistent chronic inflammation, and reduced perfusion^41^. Meanwhile, vascular endothelial dysfunction may be a common pathophysiology between ED and IBD^6^. Our study found that CD caused ED, while ulcerative colitis did not. Some previous IBD-related MR studies also had similar results. Li et al. performed an MR study and found a causal relationship for CD-psoriasis^42^. Similarly, an MR study by Freuer et al. found that CD and not UC are responsible for the causal effect of IBD on psoriasis^43^. However, these studies have yet to explore the reasons for this difference. In contrast, Shi et al. performed an MR study between IBD and celiac disease^44^. They described specific genes and dietary habits that may help to explain why only CD had a causal effect on celiac disease. Hence, genetic differences may be the main reason UC and CD have different properties. Of course, this speculation needs further research.

Our study also has some deficiencies. Firstly, this was a study based on European populations. Therefore, the results may not be generalizable to other regions. Secondly, there may be mediating factors in this causal relationship. However, we did not run mediation analyses. Thirdly, we could not perform an ED subtype analysis due to insufficient relevant data. Fourthly, the overlapping subgroups ratio was unclear in the study sample, which may affect results.

In conclusion, our study found a causal association between IBD and ED. IBD and its subtype CD may lead to ED, while UC did not cause ED. Nonetheless, further research was needed to explore the potential mechanism of the causative relationship between IBD and ED.

## Data Availability

All datasets were publicly available (https://gwas.mrcieu.ac.uk/). Further inquiries can be directed to the corresponding author.
